# Supplementary material for: Virus and viroid diversity in hops, investigating the German hop virome
Source: PLoS One. 2025 Aug 7;20(8):e0329289. doi: 10.1371/journal.pone.0329289 (PMC12331113; doi:10.1371/journal.pone.0329289)
Supplement: Attachment — Submitted filename: Rebuttal letter.docx [file pone.0329289.s008.docx]

Rebuttal Letter

Dear Dr. Uludag,

Many thanks for the feedback provided by you and the reviewers. Reviewer 1 addressed an interesting point. The reason for the low number of reads after QC is based on technical difficulties at the commercial sequencing provider. They had problems with the library preparation; to compensate their problems, we were provided with substantial more reads per library than requested. However, most of these reads were duplicated which were removed during QC. We have clarified this within the manuscript as well as adding additional information to S Table 4. A point-by-point address to the comments is attached below.

We have provided the revised manuscript in both track-change and clean version as well as revised S Table 4.

Many thanks to reviewer 1 for spotting this issue and helping us to clarify our analyses!

After the submission of our manuscript, a political change of government led to a new name for one of our funders. The correct citation should read:

“The project was supported by funds of the Federal Ministry of Agriculture, Food and Regional Identity (BMLEH) based on a decision of the Parliament of the Federal Republic of Germany via the Federal Office for Agriculture and Food (BLE) under the innovation support programme (Grant number 2818714B19).

The sequencing was funded by the Euphresco projects: VirusCurate 2019-E-312: Using High Throughput Sequencing to gain insights from virus collections and strengthening the infrastructure of Plant Virus Collections and 2020-A-347: Baseline studies on virus reservoirs.“

Whilst the text is correct in the manuscript, the online submission tool refused to accept the changes. The editorial office confirmed, that the changes could only be implemented upon acceptance of the manuscript, just to clarify this issue.

With kindest regards,

Heiko Ziebell

Ali Pasha

Gritta Schrader

Reviewer #1:

On more thing needs to be addressed, in the supplementary table S4, number of reads after QC is very low, which is unexpected. Did you maybe preformed host plant genome subtraction and then you reported the number of reads after that? If that is true please state it in the table and in the manuscript where you describe bioinfo pipeline. If this is not the case, please re-check the data you provided in the supplementary table S4, because it is really suspicious that after QC sometimes you have only about 5% of reads left, this shouldn't be the case if the sequencing went well which based on your other presented data was logical.

We also noticed the low number of reads in several datasets after quality control, as presented in S4 Table. We would like to clarify that we did not perform host plant genome subtraction during the data analysis. The low number of retained reads after quality control is can be attributed to two factors:

1- Duplicate reads from library re-sequencing:

As stated in the manuscript, we initially requested that the service provider to generate approximately 10 million paired-end reads per library. According to the service provider, in some cases, fewer reads were obtained initially, so the service re-sequenced the same library to reach the requested number. However, this re-sequencing introduced a high number of technical duplicate reads, which increased the total number of raw reads artificially.

We investigated this issue by analyzing some of our datasets in Geneious Prime with the Dedupe tool vs. 38.84 “Duplicate Read Remover” using the default settings. The results confirmed the presence of a substantial number of duplicate reads, which were subsequently filtered out during normalization with BBNorm alongside other low-quality reads with a Q-score below 20.

2- The implementation of virus/viroid enrichment method:

We performed virus and viroid enrichment (M&M). This method reduces host and microbial RNA that present in the sample. Although this method improves the detection of viruses and viroids, it results in lower total RNA yields. Sequencing providers sometimes misinterpret the low RNA yields as poor quality, so they repeated library sequencing without taking the enrichment strategy into account, and this happened in our case (clarified by the service provider AFTER we obtained the raw reads).

We used only the acquired reads after QC for downstream analysis and found them to be sufficient for detection of partial or full genomes of viruses and viroids. In addition, we were also able to identify other microorganisms and plant sequences. A new column containing the number of “obtained contigs” has been added to S4 Table.

Reviewer #2:

The manuscript PONE-D-25-26477-R1, entitled "Virus and viroid diversity in hops: Investigating the German hop virome", has been revised according to the reviewers' suggestions. I have no further comments and therefore recommend that the revised manuscript be accepted for publication.

Thank you very much for revising our manuscript - we appreciate your valuable feedback.
